# Cost-Utility Analysis of a pre-, peri- and postoperative rehabilitation pathway versus usual care in patients undergoing lumbar fusion surgery

**DOI:** 10.1016/j.bas.2025.104221

**Published:** 2025-02-26

**Authors:** Liedewij Bogaert, Olivier Nachtergaele, Tinne Thys, Peter Van Wambeke, Lotte Janssens, Thijs Willem Swinnen, Lieven Moke, Sebastiaan Schelfaut, Joost Dejaegher, Sieglinde Bogaert, Koen Peers, Ann Spriet, Wim Dankaerts, Simon Brumagne, Bart Depreitere

**Affiliations:** a Department of Physical and Rehabilitation Medicine, University Hospitals Leuven, Leuven, Belgium; b REVAL Rehabilitation Research Center, Hasselt University, Diepenbeek, Belgium; c Department of Rehabilitation Sciences, KU Leuven, Leuven, Belgium; d Department of Orthopaedics, University Hospitals Leuven, Leuven, Belgium; e Institute for Orthopaedic Research and Training (IORT), Department of Development and Regeneration, KU Leuven, Leuven, Belgium; f Department of Neurosurgery, University Hospitals Leuven, Leuven, Belgium; g Department of Movement Sciences, KU Leuven, Leuven, Belgium

**Keywords:** Lumbar fusion surgery, Economic evaluation, Rehabilitation, Cost-utility, Case manager, Indirect costs

## Abstract

**Introduction:**

The REACT trial demonstrated that a pre-, peri- and postoperative rehabilitation pathway (i.e. REACT rehabilitation) was associated with greater improvements in disability, back pain, and return-to-work rate, compared with usual care, after lumbar fusion surgery (LFS).

**Research question:**

To assess the potential cost-utility of the REACT rehabilitation relative to usual care in patients undergoing LFS for degenerative conditions.

**Materials and methods:**

A cost-utility analysis over a six-month time horizon was conducted using data from the REACT trial from the perspective of the Belgian healthcare system. A secondary analysis from a societal perspective included indirect costs associated with productivity losses. Probabilistic sensitivity analysis evaluated uncertainty. Primary outcomes were differences in costs, quality-adjusted life years (QALY), and incremental cost-effectiveness ratio (ICER).

**Results:**

The main analysis included 72 patients (mean age 55.1 years [SD 14.1], 59.7% female). The REACT rehabilitation reduced outpatient medical costs (p < 0.0001) and indirect costs (p < 0.0001), with a trend toward lower hospitalization costs (p = 0.07), despite higher rehabilitation costs (p = 0.002). There was no significant QALY improvement. The resulting ICER of −87,762.78€/QALY indicated that REACT rehabilitation was more effective and less costly than usual care. Probabilistic sensitivity analysis revealed a high probability of being cost-effective (92.8%).

Secondary analysis confirmed the cost-utility of REACT rehabilitation when including indirect costs.

**Discussion and conclusion:**

In this cost-utility analysis, the REACT rehabilitation was cost-effective compared to usual care for patients undergoing LFS. Although the REACT rehabilitation did not significantly enhance QALY or decrease total direct costs, it significantly reduced indirect costs, which outweighed direct costs in patients undergoing LFS.

## Highlights

• REACT rehabilitation has a high probability of being cost-effective in Belgium.
• REACT rehabilitation comprises 7% of total direct costs after lumbar fusion surgery.
• Indirect costs largely exceed direct costs associated with lumbar fusion surgery.
• REACT rehabilitation reduces indirect costs significantly compared to usual care.

## 1 Introduction

Lumbar fusion surgery (LFS) is a common procedure for the treatment of degenerative conditions of the lumbar spine(Reisener et al., 2020). Over the past decades, rehabilitation for LFS has undergone a paradigm shift, moving away from immobilization to safeguard the surgical construct, towards early mobilization(Debono et al., 2021) and multimodal early rehabilitation interventions that aim to improve patient functionality (Bogaert et al., 2022). The beneficial effect of rehabilitation on the clinical outcomes within six months following LFS has been demonstrated in several randomized controlled trials(Bogaert et al., 2022), however, the rehabilitation protocols showed significant variability between studies. Therefore, a pre-, peri- and postoperative rehabilitation pathway (i.e. REACT rehabilitation pathway) was recently developed, combining meta-analytic evidence and consensus from a Delphi process, to provide uniform guidance for patients and healthcare providers(Bogaert et al., 2022, 2023). This REACT rehabilitation pathway has been shown to reduce back pain and disability, and increase the return-to-work rate after LFS compared to usual care(Bogaert et al., 2025).

Despite the growing recognition of the clinical benefits of rehabilitation in patients undergoing LFS, the economic implications have remained relatively underexplored(Burgess et al., 2019). Although some studies have touched upon the economic aspects, the number of such studies is still limited.

Given the finite nature of healthcare resources, the crucial question of "value for money" inevitably arises when considering the reimbursement of any intervention(Cleemput et al., 2012). Timely cost-utility analyses serve as a valuable tool for policy makers, healthcare providers, patients, and the public to understand the economic impact of rehabilitation interventions.

Therefore, the main aim of this study was to assess the cost-utility of a pre-, peri- and postoperative rehabilitation pathway (i.e. REACT rehabilitation pathway) compared to usual care for patients undergoing one- and two-level LFS for degenerative conditions and adult isthmic spondylolisthesis, over a six-month time horizon. Additionally, differences in direct and indirect costs and utilities will be assessed over this time horizon.

## 2 Methods

A Cost-Utility Analysis (CUA) from the perspective of the Belgian healthcare system was conducted for a six-month time horizon (from the day before surgery to six months postoperatively). Primary outcomes were incremental costs, incremental quality-adjusted life-years (QALYs), and the resulting incremental cost-effectiveness ratio (ICER). All analyses were conducted using R software(R Core Team, 2020). This study was approved by the Ethics Committee Research UZ/KU Leuven. All patients provided informed consent prior to participation. The study is reported in accordance with the Consolidated Health Economic Evaluation Reporting Standards (CHEERS)(Husereau et al., 2022).

### 2.1 Data collection

The present study included intention-to-treat data from the REACT trial, which was a single-center, nonrandomized controlled trial of 72 patients aged between 18 and 75 years undergoing a first-time one- and two-level LFS for degenerative conditions or (adult) isthmic spondylolisthesis(Bogaert et al., 2025). Patients were allocated in a time-dependent manner based on the scheduled date of surgery. Thirty-six patients were assigned to usual care (i.e. treatment as recommended by the treating surgeon or other healthcare providers). After the implementation of the REACT rehabilitation pathway, 36 consecutive patients were allocated to the REACT pathway.

The REACT rehabilitation pathway included the following key components(Bogaert et al., 2025).

• Prehabilitation with a preoperative physiotherapeutic and case manager assessment to identify potential risk factors for rehabilitation and to address these accordingly (e.g. optimize rehabilitation plan)
• Early mobilization and avoidance of unsubstantiated postoperative restrictions
• Early postoperative active physiotherapy and empowerment (or interdisciplinary treatment for patients with high-risk factors)
• Motivation towards an early return to activity and work
• Case manager guidance in the pre-, peri-, and postoperative rehabilitation
• Uniform and positive communication from the interdisciplinary team, promoting an early return to activity and work

#### 2.1.1 Health state utility values

Health-related quality of life was assessed by the EuroQol 5-Dimension 3-Level (EQ-5D-3L) instrument preoperatively and at four days, six weeks, three months, and six months postoperatively. Utility scores were computed from the EQ-5D-3L data using the Belgian valuation set, where a score of 1 corresponds to a state of perfect health and a score of 0 corresponds to death. We applied multiple imputations using predictive mean matching to address missing values (5.1% in the control group and 1.4% in the REACT group across all time points). Quality-adjusted life years (QALYs) were computed from the utility scores by calculating the time-weighted area under the curve(Devlin and Janssen, 2020).

#### 2.1.2 Costs

Per convention for the health care system perspective, and as recommended in the Belgian guidelines for health economic analysis(Cleemput et al., 2012), only direct medical costs were computed in the main analysis, including surgery, hospitalization, technical investigation, rehabilitation, and clinic fees. All inpatient and outpatient cost data were sourced from the hospital's financial database. Costs for physiotherapy sessions were obtained from the RIZIV/IMANI (using the defined rate for 2022)(RIZIV/IMANI, 2024). The average cost per person for an interdisciplinary outpatient rehabilitation program was calculated based on the cost data from the hospital's financial database. All costs were adjusted for the 2022 financial year using the health index for Belgium (Appendix A).

A secondary analysis from a societal perspective included indirect costs resulting from work absenteeism. Productivity costs were valued using the Human Capital Approach, assuming that each day of absence corresponds to the average national labor cost per day(Cleemput et al., 2012). The return-to-work date and the corresponding work percentage (and subsequent dates and work percentages in case of gradual work resumption) were used to calculate the total number of postoperative sick leave days. Productivity costs were then calculated by multiplying the total number of postoperative sick leave days by the average daily labor cost in Belgium (estimated at €227.1 for costing year 2022) (Cleemput et al., 2012; Eurostat, 2024). Only patients who were part of the workforce (working or on sick leave) were included in this secondary analysis (N = 43). Notably, only costs incurred up to six months postoperatively were included.

### 2.2 Analyses

#### 2.2.1 Statistical analysis

EQ-5D-3L utility scores, costs, and postoperative sick leave days were reported as means with standard deviations, and differences between groups were compared using a two-sample *t*-test with a significance level of α = 0.05.

#### 2.2.2 Cost-utility analysis

The ICER was calculated by dividing the incremental costs by the incremental utilities. Thereby, the ICER represents the cost per QALY gained. Cost and utility input parameters were derived directly from the REACT trial. Given the time horizon of six months, no annual discounting was applied.

$$\frac{{\text{Cost}}_{\text{REACT}\;}–\;{\text{Cost}}_{\text{Control}}}{{\text{Utility}}_{\text{REACT}\;}–\;{\text{Utility}}_{\text{Control}}}=\frac{\Delta \text{Cost}}{\Delta \text{Utility}}=\text{ICER}$$

Nonparametric bootstrapping was performed with 10,000 replications and subsequent calculation of ICER values, allowing for an estimation of the overall uncertainty surrounding the incremental cost-effectiveness output. This method facilitated the construction of both a cost-effectiveness plane and a cost-effectiveness acceptability curve (van Dongen et al., 2014). Note that Belgium does not have a fixed ICER threshold value for determining the cost-effectiveness of interventions. Thereby, the willingness-to-pay threshold was set at the gross domestic product (GDP) per capita of Belgium (€47,430/QALY in 2022), which is consistent with the threshold often used in other high-income countries and with the World Health Organization guidelines(Eurostat, 2023; Pichon-Riviere et al., 2023). The cost-effectiveness acceptability curve visualized the probability that the REACT rehabilitation pathway was cost-effective for a range of willingness-to-pay threshold values.

A secondary analysis was performed from a societal perspective, considering both direct and indirect costs, and included patients in the workforce. Given the dominant contribution of inpatient costs to the overall direct medical costs, an additional secondary analysis was performed that considered direct medical costs resulting from outpatient care and rehabilitation services, yet excluded the inpatient costs.

## 3 Results

### 3.1 Utilities and costs

The REACT study included 72 patients, of whom 36 were allocated to usual care (mean age 51.1 [SD 13.3], 56% female) and 36 were allocated to the REACT rehabilitation pathway (mean age 59.0 [SD 14.1], 64% female). Health-related quality of life improved over time in both groups, but no significant differences were observed between groups (Table 1).

**Table 1:** Health-related quality of life, as measured by the EuroQol 5-Dimension 3-Level (EQ-5D-3L) with Belgian valuation.

	Group	Mean (Standard Deviation)				
		Baseline	4 days	6 weeks	3 months	6 months
**EQ-5D-3L**	**REACT**	0.43 (0.22)	0.47 (0.21)	0.66 (0.23)	0.72 (0.16)	0.77 (0.18)
	**Control**	0.41 (0.22)	0.40 (0.18)	0.59 (0.18)	0.69 (0.17)	0.72 (0.23)
	***P value***	*0.66*	*0.14*	*0.17*	*0.36*	*0.29*

After six months, patients in the REACT group had significantly fewer postoperative sick leave days (mean 95 days [SD 47.8]) than patients in the usual care group (mean 166.9 days [SD 27.6], p < 0.0001).

Data from the hospital's financial database revealed that inpatient direct costs (i.e. hospitalization expenses) constituted the dominant portion of total direct costs in both the REACT (92.2%) and control group (94.8%) (Table 2). While there was no significant difference in hospitalization costs between the groups, a trend toward lower costs was observed in the REACT group (p = 0.07). Rehabilitation costs accounted for 7.0% and 3.1% of total direct costs in the REACT and control group, respectively. Outpatient medical costs, such as imaging and surgical consultations, were minor components of the direct cost profile, constituting 0.8% of the total costs in the REACT group and 2.1% in the control group.

**Table 2:** Total direct medical costs (€) per group and per patient (N = 72), and total indirect costs (€) per patient who is part of the working force due to productivity loss (only patients who were part of the workforce (working or on sick leave) were included in this secondary analysis, N = 43).

	REACT (N = 36)	Control (N = 36)	P value
TOTAL DIRECT COSTS	**€ 474,660.64**	**€ 555,492.67**	
*= Mean total direct medical costs per patient [SD]*	***€ 13,185.02 [4514.97]***	***€ 15,430.35 [6694.08]***	***0.10***

Inpatient cost (total)	**€ 437,598.46**	**€ 526,557.26**	
*= Mean total inpatient costs per patient [SD]*	*€ 12,155.51 [4454.28]*	*€ 14,626.59 [6725.75]*	*0.07*
Nursing days	€ 201,162.14	€ 269,823.02	
Medical Care (surgery, imaging, medication, …)	€ 236,436.32	€ 256,734.24	

Outpatient cost (total)	**€ 3678.36**	**€ 11,815.19**	
*= Mean total outpatient costs per patient [SD]*	*€ 102.18 [112.5]*	*€ 328.20 [182.97]*	*<0.0001*
Consultation fee	€ 1269.40	€ 5771.65	
Imaging of lumbar spine/full spine	€ 1933.04	€ 4767.29	
Other technical investigations (imaging of related problems e.g. cervical, pelvic; electromyography; lab tests; …)	€ 475.92	€ 1276.25	

Rehabilitation cost (physiotherapy, interdisciplinary) (total)	**€ 33,383.82**	**€ 17,120.22**	
*= Mean total rehabilitation costs per patient [SD]*	*€ 927.33 [465.77]*	*€ 475.56 [697.34]*	*0.002*
Case manager consultations	€ 5421.60	€ 0	
Preoperative physiotherapeutic intake	€ 832.00	€ 0	
Physiotherapy sessions	€ 22,932.00	€ 12.922.00	
Interdisciplinary rehabilitation	€ 4198.22	€4198.22	

	**REACT (N = 16)**	**Control (N = 27)**	**P value**
TOTAL INDIRECT COSTS			
*= Mean total indirect costs per patient [SD]*	***€ 21,564.24 [10,844.09]***	***€ 38,036.18 [6195.40]***	***<0.0001***

**Fig. 1:**
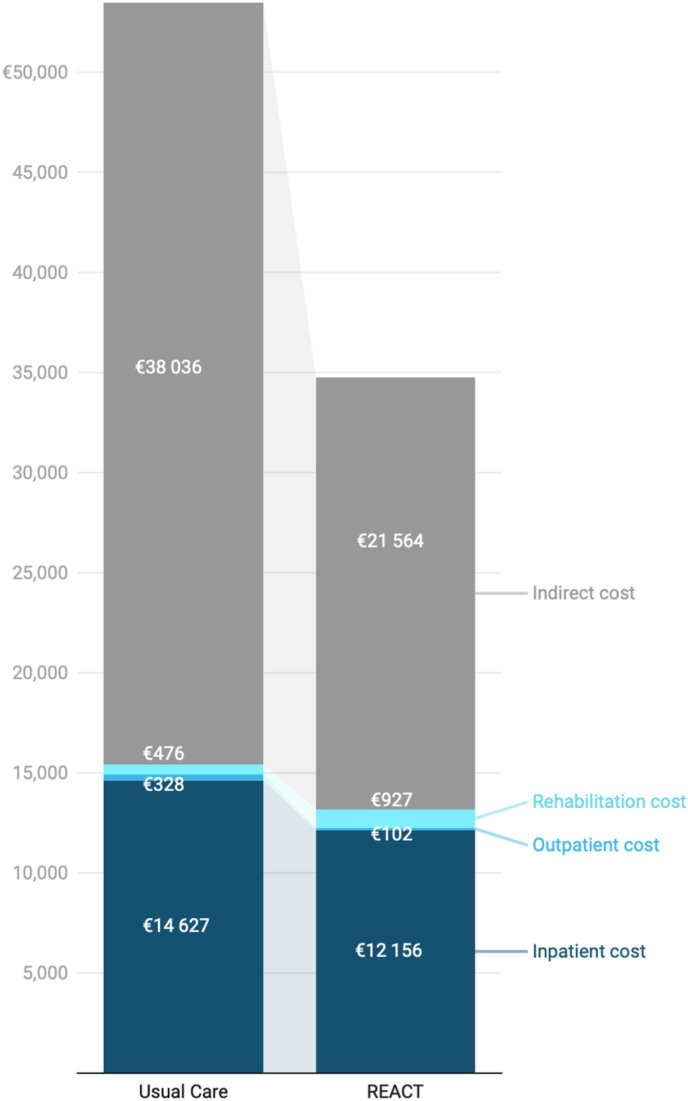
Summary of the mean overall costs (€) per person, including direct medical costs (dark to light blue, including inpatient medical costs, outpatient medical costs and rehabilitation costs) and indirect societal costs due to productivity losses (gray).

Notably, indirect costs due to productivity loss far exceeded direct costs in both groups (Fig. 1). The REACT group exhibited significantly lower indirect costs per patient than the control group (Table 2).

### 3.2 Cost-utility analysis

#### 3.2.1 Primary analysis

In the primary analysis, the REACT rehabilitation was the dominant treatment option at an ICER of −87,762.78€/QALY (Table 3).

**Table 3:** Results of the primary and secondary cost-utility analyses.

	REACT	Usual Care
Main analysis		

ICER, €/QALY	**−87,762.78**	**Dominated**
Mean [SD] total (direct) cost per patient, €	€ 13,185.02 [4514.97]	€ 15,430.35 [6694.08]
Mean [SD] QALYs per patient, QALY	0.345 [0.078]	0.319 [0.075]

Secondary analysis: *direct and indirect costs*		

ICER, €/QALY	−561,588.70	Dominated
Mean [SD] total (direct and indirect) cost per patient, €	€ 34,630.60 [11,411.83]	€ 52,454.16 [7341.62]
Mean [SD] QALYs per patient, QALY	0.345 [0.052]	0.313 [0.082]

Additional secondary analysis: *direct medical costs (excluding inpatient costs)*		

ICER, €/QALY	8823.58	NA
Mean [SD] total (outpatient and rehabilitation) cost per patient, €	€ 1029.51 [440.45]	€ 803.76 [787.77]
Mean [SD] QALYs per patient, QALY	0.345 [0.078]	0.319 [0.075]

ICER: incremental cost-effectiveness ratio; QALY: quality-adjusted life-years; SD: standard deviation.

During the six-month follow-up, usual care resulted in an average total cost of € 15,430.35 per patient, compared with € 13,185.02 per patient receiving the REACT rehabilitation pathway, resulting in a difference of €2245.33 per patient (95% CI, −4935.95 to 445.28). A mean of 0.319 and 0.345 QALYs were accrued in the usual care group and REACT group, respectively, with a between-group difference of 0.0256 QALYs (95% CI, −0.010 to 0.062).

**Fig. 2:**
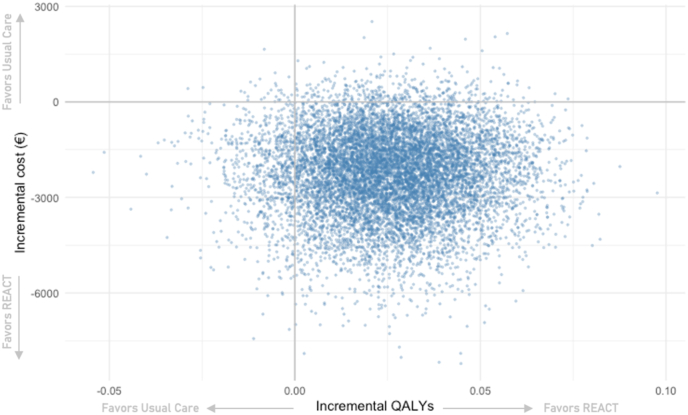
The cost-effectiveness plane for REACT rehabilitation compared to usual care for lumbar fusion surgery, based on 10,000 replications of ICERs after nonparametric bootstrapping.

**Fig. 3:**
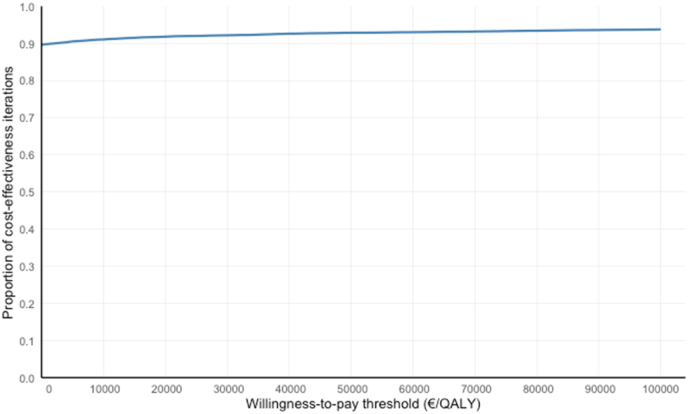
Cost-effectiveness Acceptability Curve of REACT rehabilitation for lumbar fusion surgery.

Across the 10,000 replications of the nonparametric bootstrapping, a majority (89.4%) resulted in an ICER in the southeast quadrant of the cost-effectiveness plane, where REACT dominated usual care (Fig. 2). Overall, the ICER values remained below than the willingness-to-pay threshold of €47,430/QALY in 92.8% of the simulations. The cost-effectiveness acceptability curve showed that REACT was more cost-effective than usual care in the majority of simulations (range from 89.6% to 93.7%) across a willingness-to-pay threshold range of €0/QALY to €100 000/QALY (Fig. 3).

#### 3.2.2 Secondary analysis

In the secondary analysis from a societal perspective, which includes both direct and indirect costs, the REACT rehabilitation program was associated with significantly lower total costs per patient within the workforce of €34,630.60 compared to €52,454.16 for usual care. This represents a difference of €17,823.56 (95% CI, −24,599.71 to −11,047.41) (p < 0.001). The REACT rehabilitation strategy remained the more cost-effective option compared to usual care, with an ICER of −561,588.70 €/QALY.

An additional analysis was conducted from a healthcare system perspective, considering only outpatient and rehabilitation costs, thereby excluding inpatient costs. The reduction in mean outpatient cost per patient did not offset the higher rehabilitation costs in the REACT group, resulting in a mean incremental cost of € 225.75 per patient (95% CI, −80.00 to 531.48). Consequently, the REACT rehabilitation strategy was associated with an ICER of € 8823.58/QALY. Nonparametric bootstrapping with 10,000 iterations demonstrated that the ICER was situated in the northeast quadrant of the cost-effectiveness plane in 86.1% of iterations and in the southeast quadrant 6.4% of iterations (Appendix B). The probability of cost-effectiveness, considering the willingness-to-pay threshold of Belgian GDP per capita, remained high (95.3%).

## 4 Discussion

The cost-utility analysis from a healthcare system perspective demonstrated that the REACT rehabilitation pathway was the dominant strategy at an ICER of −87,762.78€/QALY. This suggests that REACT rehabilitation is more effective and less costly than usual care. The probabilistic sensitivity analysis results reinforced this finding, and the REACT rehabilitation remained cost-effective across a range of willingness-to-pay thresholds.

A prespecified secondary analysis from a societal perspective, considering both direct and indirect costs, in the patients of the REACT trial who were part of the workforce confirmed the finding that REACT rehabilitation was cost-effective, at an ICER of −561,588.70€/QALY. This higher cost-effectiveness was driven by a significant reduction in sick leave and thereby in indirect costs in patients who are part of the working force.

Despite the growing recognition of the importance of rehabilitation after lumbar fusion surgery, previous economic evaluations of rehabilitation for LFS remain scarce and show inconsistent results. For instance, preoperative education has been proposed to improve the quality of life of patients undergoing LFS while maintaining cost neutrality(Rolving et al., 2016), and preoperative exercise therapy with early postoperative rehabilitation has been suggested to reduce direct and indirect costs in this population (Nielsen et al., 2008). On the other hand, certain strategies, such as case management and initiating exercise rehabilitation at six weeks instead of twelve weeks after LFS, have raised concerns about their cost-effectiveness(Oestergaard et al., 2013, 2020).

In this study, most of the total medical costs (>90%) were attributed to inpatient care, which is consistent with previous research showing that more than 80% of the total costs for hip and knee arthroplasty are attributed to inpatient care (Crawford et al., 2021). The mean REACT rehabilitation cost per patient remained below €1000, constituting 7.0% of the total medical costs per patient. This suggests that the REACT rehabilitation pathway is a relatively low-cost intervention. When only outpatient healthcare services and rehabilitation costs were considered, the REACT rehabilitation was associated with an ICER of €8823.58/QALY, which is highly comparable to the economic analysis by Paulsen et al., who found that intensive physiotherapy after lumbar disc surgery was associated with an ICER of €10,085.7/QALY(Paulsen et al., 2020).

While the decrease in outpatient medical care expenses might appear minor given their smaller share of overall costs, it is important to emphasize that average consultation fees within the usual care group were four times higher compared to those in the REACT group. Similarly, lumbar spine imaging costs were twice as high in the usual care group, thereby suggesting that the REACT rehabilitation might reduce the burden on medical services.

Furthermore, the average costs associated with work absenteeism per patient exceeded the total medical costs in both groups. For patients undergoing LFS with usual care, the costs attributed to work absenteeism were more than double the overall medical costs.

This cost-utility analysis addresses an important gap in the existing literature by providing economic insights into the relative costs associated with a pre-, peri-, and postoperative rehabilitation pathway in relation to total medical costs associated with LFS. This analysis may provide economic justification for the role of rehabilitation in LFS care, where its use has predominantly been supported by clinical evidence thus far(Bogaert et al., 2022; Madera et al., 2017; Greenwood et al., 2016). Additionally, considering that certain components, such as case manager guidance, are currently not reimbursed by the Belgian healthcare system, this study could serve as a reference for healthcare policy makers when considering efficient resource allocation.

### 4.1 Limitations

The results of this study should be interpreted in light of several limitations. First, the outpatient physiotherapy sessions were the only expenses not directly obtainable from the hospitals' financial database. The unit cost per physiotherapy session was based on the convention rate set by the Belgian National Institute for Health and Disability Insurance. It is important to note that not all physiotherapists in Belgium adhere to this convention, suggesting that the actual cost of physiotherapy may be possibly a little higher than the estimated figure. Second, it should be noted that these results are based on the Belgian healthcare system and may not be directly generalizable to countries with different healthcare structures and cost estimates. Third, the case manager in this study was a specialist in physical and rehabilitation medicine. Additional contacts via e-mail or telephone were not included in this economic analysis and were considered an integral part of the follow-up inherent to a medical consultation. However, the time required for these contact moments, as well as any interdisciplinary discussions, could potentially exceed the time typically involved in a standard medical consultation, which may result in slightly higher rehabilitation costs. The costs associated with case manager follow-up may vary slightly if another healthcare provider, such as a physiotherapist or nurse practitioner, assumes the case manager role. Fourth, costs related to analgesics, general practitioner visits, and non-related healthcare were not included due to data limitations, possibly leading to a slight underestimation of total direct costs. Fifth, the short six-month time horizon limits the ability to draw conclusions regarding the long-term cost-utility of REACT rehabilitation for patients undergoing LFS. However, considering that inpatient direct costs (i.e., hospitalization expenses) constituted the majority of total direct costs, and that no rehospitalizations were observed within the first postoperative year of follow-up, it is unlikely that our conclusion would change by extending the time horizon. Moreover, given that return-to-work rates remained significantly higher in the REACT group beyond six months postoperatively(Bogaert et al., 2025), it is likely that indirect costs would surpass direct costs to an even greater extent. Finally, the secondary analysis only accounted for indirect costs due to work absenteeism. Other indirect costs, such as work presenteeism and unpaid work, also contribute to the total cost of LFS.

## 5 Conclusions

In this cost-utility analysis, the REACT rehabilitation pathway was considered cost-effective compared with usual care in patients undergoing LFS over a six-month time horizon. The REACT rehabilitation seems to be a low-cost intervention, contributing 7% of the total direct medical costs. Although the REACT rehabilitation did not significantly improve health utilities or decrease total direct medical costs, it substantially reduced indirect costs due to productivity losses. This is important as indirect costs were found to largely exceed direct costs in patients undergoing LFS. Additional economic evaluations are necessary to draw definitive conclusions regarding the long-term cost-utility and other sources of indirect costs.

## Declaration of competing interest

The authors declare the following financial interests/personal relationships which may be considered as potential competing interests: Liedewij Bogaert reports financial support was provided by Research Foundation 10.13039/501100011878Flanders. If there are other authors, they declare that they have no known competing financial interests or personal relationships that could have appeared to influence the work reported in this paper.

## Figures and Tables

**Table:** 

Year	Health index	HI
2016	83.63001	1.1637
2017	85.41006	1.145899
2018	87.16573	1.128343
2019	88.41746	1.115825
2020	89.07584	1.109242
2021	91.24604	1.08754
2022	100	1
2023	103.552	0.96448

**Figure:**
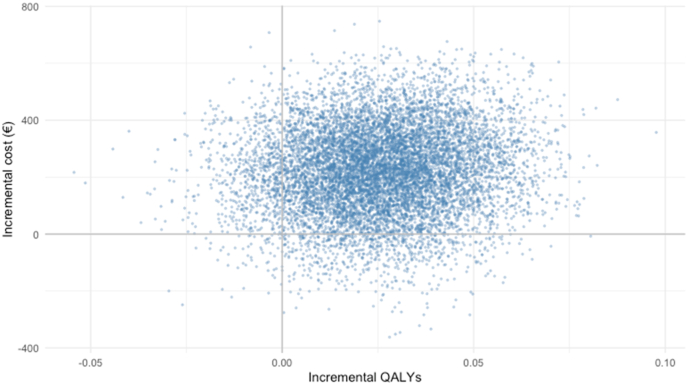


**Figure:**
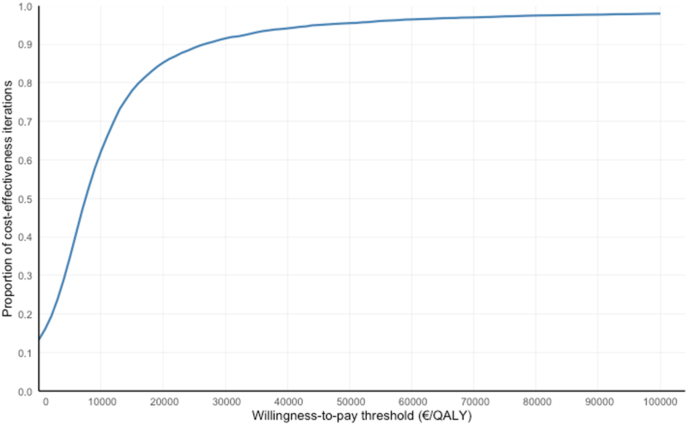


## Appendix B Sensitivity analysis for secondary analysis from societal perspective

The cost-effectiveness plane and probability of being cost-effective over a range of willingness-to-pay thresholds, based on the sensitivity analysis using non-parametric bootstrapping with 10,000 iterations for the additional secondary analysis of ICER considering direct medical costs (excluding inpatient costs).
